# Editorial: Impact of the gut microbiota on cardiovascular medicine

**DOI:** 10.3389/fmed.2022.939890

**Published:** 2022-07-13

**Authors:** Arash Haghikia, Thijs E. van Mens, Giulia Pontarollo, Christoph Reinhardt

**Affiliations:** ^1^Department of Cardiology, Charité-Universitätsmedizin Berlin, Campus Benjamin Franklin, Berlin, Germany; ^2^DZHK (German Center of Cardiovascular Research), Partner Site Berlin, Berlin, Germany; ^3^Berlin Institute of Health (BIH) at Charité - Universitätsmedizin Berlin, Berlin, Germany; ^4^Department of Vascular Medicine, Amsterdam Cardiovascular Sciences, Amsterdam Reproduction and Development, Amsterdam University Medical Center, University of Amsterdam, Amsterdam, Netherlands; ^5^Center for Thrombosis and Hemostasis (CTH), University Medical Center Mainz, Johannes Gutenberg University Mainz, Mainz, Germany; ^6^German Center for Cardiovascular Research (DZHK), University Medical Center of the Johannes Gutenberg University Mainz, Partner Site Rhine Main, Mainz, Germany

**Keywords:** microbiota, cardiovascular disease, thrombosis, drug-microbiota interactions, metabolism

In recent years, accumulating evidence from translational research has highlighted the significant role of the gut microbiota and its metabolites in shaping the cardiovascular and cardiometabolic phenotypes. In particular, combined metagenomic and metabolomic approaches have contributed to our understanding of how intestinal microbial metabolism of dietary nutrients impact metabolic pathways and cardiovascular disease risk (1, 2).

A number of metaorganismal circuits have been reported to modulate different aspects of cardiovascular and cardiometabolic conditions, such as arterial hypertension and hypertensive end organ damage (3, 4), lipid metabolism and atherosclerosis (5, 6), glucose metabolism (7), platelet activation and thrombus formation (8–10), and ischemic heart disease (11). Remarkably, recent studies suggest that gut microbial composition also impacts on the efficacy of cardiovascular pharmacotherapy (12, 13). Targeting of distinct gut microbial pathways may help to establish novel strategies in preventing cardiometabolic and cardiovascular diseases.

In the current article collection on the *Frontiers in Medicine* Research Topic “*Impact of the Gut Microbiota on Cardiovascular Medicine*” we highlight research and review articles presenting novel insights into the interaction between the gut microbiota and cardiometabolic and cardiovascular (patho-)physiology.

Lang et al. provide novel evidence of how manipulation of plasma lipid levels modulates the gut microbiome. In particular, manipulated plasma lipid levels of non-human primates using dietary and pharmacological techniques, and characterized the microbiome using 16S rRNA gene sequencing. Their study showed that high-fat diets significantly reduced alpha diversity (Shannon) and the Firmicutes/Bacteroidetes ratio compared to chow diets, even when the diets had different compositions and were applied in different orders. Pharmacological manipulation of plasma lipid levels through parenteral means however, using antisense oligonucleotides, did not affect the microbiome composition. Moreover, they demonstrated that liver X receptor (LXR) agonist treatment shifted the microbiome. Fifteen genera increased with the LXR agonist, while seven genera decreased. For example, Pseudomonas increased upon LXR agonist treatment and was negatively correlated to deoxycholic acid, cholic acid, and total bile acids while Ruminococcus was positively correlated with taurolithocholic acid and taurodeoxycholic acid. Most bile acids identified in the feces significantly decreased in response to LXR agonist treatment, and total bile acids were reduced by 62%. These results emphasize that bile acids, derived in part from plasma lipids, are likely responsible for the indirect relationship between plasma lipid levels and the microbiome. Since bile acids are toxic to bacteria, they appear to considerably shift the microbiome composition. Thus, the indirect relationship between microbiome and plasma lipid levels is most likely mediated by bile acids.

In another study Gesper et al. demonstrate a functional link between the gut-derived metabolite indole-3-propionic acid and mitochondrial function in cardiomyocytes and how this metabolite alters cardiac function. First, the authors screened 25 microbial metabolites in a live-cell metabolic assay using HL-1 cardiomyocytes. They identified indole-3-propionic acid, a microbial tryptophan derivative, as a modulator of mitochondrial function. While acute treatment induced enhancement of maximal mitochondrial respiration, chronic exposure led to mitochondrial dysfunction. In isolated perfused mouse hearts, indole-3-proprionic acid was shown to dose-dependently improve cardiac contractility. Since indole-3-propionic acid also reduced maximal respiration in cultured endothelial and hepatocyte derived cellular carcinoma cells, the revealed effect of this microbiota-derived tryptophan derivative is not restricted to cardiac tissue. Cumulatively, their results indicate a direct impact of microbial metabolites on cardiac physiology.

Ding et al. present the study design of a prospective observational case–control study to analyze the variation in the intestinal microflora and metabolites in patients undergoing cardiac surgery with cardiopulmonary bypass (CPB) and to observe the outcomes of patients with routine clinical interventions. This study is of particular interest as in the critical care unit, most common clinical interventions such as enteral feeding, proton-pump inhibitors, systemic catecholamines and systemic antibiotics change the intestinal microbiome composition. Since in critically ill patients, changes in the intestinal flora and metabolites co-occur with systemic inflammatory reactions and often bacterial sepsis, the authors hypothesize that intestinal dysbacteriosis after CPB, enhanced by intestinal ischemia-reperfusion injury, is a possible mechanism of inflammation and that bacterial translocation contributes to the development of sepsis.

Finally, Chen et al. summarize current knowledge about antihypertensive drug-microbiota interactions and discuss how these interactions may help to develop gut microbiota-based personalized concepts for disease management, including antihypertensive response biomarkers, microbial-targeted therapies, and probiotics therapy.

These contributions shed further light into the interaction between the gut microbiome and (patho-)physiolgogical pathways in the cardiovascular system and highlight the potential of microbiota-related strategies to improve currently available diagnostic and treatment options in cardiovascular medicine.

## Author contributions

AH wrote the first draft of the manuscript. TvM, GP, and CR wrote sections of the manuscript. All authors read and approved the submitted version.

## Funding

CR is a fellow of the Gutenberg Research College at the Johannes Gutenberg-University Mainz, a scientist at the German Center for Cardiovascular Research (DZHK), Partner Site Rhine Main, and acknowledges funding from the Forschungsinitiative Rheinland-Pfalz and ReALity. AH is participant in the BIH-Charité Advanced Clinician Scientist Pilotprogram funded by the Charité – Universitätsmedizin Berlin and the Berlin Institute of Health.

## Conflict of interest

The authors declare that the research was conducted in the absence of any commercial or financial relationships that could be construed as a potential conflict of interest.

## Publisher's note

All claims expressed in this article are solely those of the authors and do not necessarily represent those of their affiliated organizations, or those of the publisher, the editors and the reviewers. Any product that may be evaluated in this article, or claim that may be made by its manufacturer, is not guaranteed or endorsed by the publisher.
